# Association between cervical conization and pregnancy outcomes: A nationwide population-based cohort study

**DOI:** 10.1371/journal.pone.0341660

**Published:** 2026-02-17

**Authors:** Woo Jeng Kim, Yong-Wook Kim

**Affiliations:** Department of Obstetrics and Gynecology, Incheon St. Mary’s Hospital, College of Medicine, The Catholic University of Korea, Seoul, Republic of Korea; Xiangya Hospital Central South University, CHINA

## Abstract

Cervical conization is the standard treatment for cervical intraepithelial neoplasia. However, its effects on pregnancy outcomes remain controversial. Using data from the Korean National Health Insurance System, this population-based retrospective cohort study evaluated the relationship between cervical conization and adverse pregnancy outcomes between 2006 and 2022. Altogether, 199,826 singleton primiparous women aged 19 years or older were included, of whom 18,602 had undergone conization prior to pregnancy. The adjusted odds ratios for obstetric complications were estimated using multivariable logistic regression. Conization was associated with an elevated risk of cervical incompetence (adjusted odds ratios [aOR] 3.15; 95% confidence interval [CI] 3.01–3.30), preterm labor (aOR 1.44), preterm premature rupture of membranes (aOR 1.67), placenta previa, gestational diabetes, and intrauterine growth restriction. The subgroup analysis revealed that women who underwent both conization and cerclage had substantially higher risks of preterm labor and preterm premature rupture of membranes than did those who did not receive cerclage. The results point to a heightened obstetric risk following cervical conization, emphasizing the need for tailored prenatal care and continued prospective investigation.

## Introduction

Cervical conization, commonly referred to as cone biopsy, is a surgical intervention designed to excise a cone-shaped segment of cervical tissue [1]. Excisional procedures, such as conization, play a key role in the diagnostic evaluation of both glandular and squamous intraepithelial abnormalities. These procedures are particularly valuable for identifying lesions suspected of early neoplastic transformation, allowing for timely identification and management of cervical cancer precursors [2]. Although cervical conization effectively treats abnormal cervical lesions, concerns have been raised regarding its potential impact on subsequent pregnancy outcomes.

Cervical excisional procedures, including conization, have been linked to impaired cervical structural integrity, which may increase the risk of obstetric complications, such as cervical insufficiency and preterm delivery, particularly when performed multiple times or at greater excision depths [3]. Multiple clinical studies, including large-scale meta-analyses and population-based cohort studies, have reported that women undergoing cervical excision procedures exhibit a higher risk of adverse pregnancy outcomes, such as second-trimester loss, preterm labor, and neonatal morbidity, than do women who have not undergone such procedures [4–9]. However, some studies failed to identify statistically significant differences or have reported only marginal increases in risk, especially when less extensive procedures are used or when potential confounders are taken into account [10–13]. Thus, although cervical conization is widely implemented in clinical practice, its effects on subsequent pregnancy outcomes remain debatable.

These conflicting findings highlight a lack of consensus in the literature and emphasize the need for broader, population-level, and methodologically robust studies to better clarify the relationship between cervical excision and reproductive health. This issue is particularly important because cervical intraepithelial neoplasia (CIN) frequently affects women of reproductive age, and excisional procedures such as conization and loop electrosurgical excision (LEEP) are widely used for its management [14]. As many patients have not yet completed childbearing at the time of diagnosis, it is essential to understand the long-term reproductive implications of cervical excision. Such knowledge can support informed clinical decision-making and improve patient counseling [9,15,16]. Accordingly, we leveraged nationwide claims data to refine risk estimates after conization and to inform counseling in clinical practice. In this study, we aimed to investigate the association between cervical conization and adverse pregnancy outcomes in a large nationwide cohort, with the goal of supporting evidence-based prenatal care and management strategies.

## Materials and methods

### Data source and study population

This retrospective cohort analysis was conducted using the Korean National Health Insurance Service (NHIS) claims database, a nationwide system that encompasses nearly the entire South Korean population under universal healthcare coverage. The database contains detailed information, including demographic data, diagnostic and procedural codes, prescription records, and the results of standardized national health screenings.

Women aged 19 years or older who gave birth between 1 January 2006 and 31 December 2022, were initially identified (n = 1,344,628). Exclusion criteria included incomplete data regarding health screenings, diagnostic evaluations, or procedural histories. After applying these criteria, only singleton primiparous women were selected, resulting in a final analytical cohort of 199,826 individuals. We restricted the cohort to primiparous singleton births to minimize confounding by prior obstetric history, which can materially influence subsequent pregnancy outcomes independent of prior conization. This design improves internal validity while reducing generalizability to multiparas. Participants were categorized based on whether they had undergone cervical conization prior to pregnancy. Conization status was determined using the national procedure codes R4261 and R4262, which represent cold knife and electrosurgical conization procedures, respectively, as recorded in the NHIS claims database before pregnancy. Altogether, 181,224 women without these procedure codes were categorized into the non-conization group, with 18,602 women in the conization group.

### Data collection

For each participant, the relevant variables were extracted from the NHIS database prior to delivery. Data were accessed for research purposes between 11 March 2024 and 18 April 2025, following IRB approval. These variables included demographic characteristics (age and body mass index), baseline comorbidities (hypertension, diabetes mellitus, hyperthyroidism, hypothyroidism, liver disease, heart disease, dyslipidemia and autoimmune disease), gynecological conditions (polycystic ovary syndrome, uterine myoma, adenomyosis, and ovarian mass), and obstetric history (missed abortion, spontaneous abortion). Induced or surgical abortions were not incorporated in the primary analyses due to inconsistent capture across the study period in claims data. Potential covariates such as antenatal genital tract infections and uterine anomalies were not incorporated because of inconsistent capture and uncertain validity in claims-level coding. Exposure to cervical conization was determined using the national procedure codes R4261 and R4262, which were recorded prior to pregnancy. Obstetric outcomes were determined using diagnostic and procedural codes as described below. Covariates for multivariate analyses were selected *a priori* based on clinical relevance and previous literature.

### Outcome measures

Obstetric outcomes were identified using the diagnosis and procedure codes from the NHIS claims database. All diagnostic codes were based on the 7^th^ edition of the Korean Classification of Diseases, which is consistent with the 10th revision of the International Classification of Diseases (ICD-10).

Gestational hypertension was defined using code O13; mild and severe preeclampsia using codes O14.0, O14.9, and O14.1; and eclampsia using code O15. Gestational diabetes mellitus (GDM) was identified using code O24.4. Placental abnormalities included placenta previa (O44.1), placenta accreta (O43.2), and placenta abruption (O45). Preterm and threatened preterm labor were identified using codes O60 and O60.0, respectively. These codes represent symptomatic diagnoses of labor rather than delivery outcomes. Preterm premature rupture of membranes (PPROM) was identified using the codes O42.00, O42.10, O42.20, and O42.90. Intrauterine growth restriction (IUGR) was identified using O36.5, threatened abortion using O20.0, retained placenta using O72.2, and polyhydramnios and oligohydramnios using O40 and O41.0, respectively.

Cervical incompetence was defined using the diagnostic codes O34 and N88. Cervical cerclage procedures were identified using procedure codes R4281, R4282, R4283, and R4284.

### Subgroup analysis

Subgroup analysis was conducted within the conization group to evaluate whether cervical cerclage modified the risk of adverse obstetric outcomes. Women in this group were further stratified according to whether the cervical cerclage procedure code was recorded before or during pregnancy. Obstetric outcomes were compared between women who underwent cerclage and those who did not. The covariates adjusted for in the subgroup analysis were consistent with those used in the primary analysis, including maternal age, body mass index (BMI), diabetes mellitus (DM), hypertension, thyroid disorders (hyperthyroidism and hypothyroidism), polycystic ovary syndrome (PCOS), and a history of missed or spontaneous abortion. We did not perform time-dependent analyses because the NHIS claims data do not reliably capture the dates of pregnancy or cerclage procedures. As the interval between the procedure and pregnancy could not be ascertained with sufficient accuracy, cerclage was not modeled as a time-dependent exposure.

### Statistical analysis

All statistical analyses were conducted using the SAS software version 9.4.1 (SAS Institute Inc., Cary, NC, USA) via remote access to the secure NHIS Big Data analysis platform. Owing to data protection policies, the analysis code is stored within the NHIS environment and cannot be shared externally. However, all variables and analytical procedures were standardized and reproducible within the platform.

Descriptive statistics were used to compare the baseline characteristics between the groups. Continuous variables were analyzed using independent t-tests, and categorical variables were assessed using the chi-squared test. Associations between cervical conization and obstetric outcomes were evaluated using multivariate logistic regression, with results expressed as adjusted odds ratios (aOR) and 95% confidence intervals (CI).

### Ethics statement

This study was exempted from full IRB review by the Institutional Review Board of the Catholic Medical Center (approval number: OC23ZISI0047) on 28 April 2023. The requirement for informed consent was waived because the study used de-identified administrative data from a national claims database and posed minimal risk to the participants. All methods were conducted in accordance with relevant guidelines and regulations.

## Results

### Baseline characteristics

Table 1 summarizes the baseline characteristics of the study population categorized according to the conization status. The mean maternal age was similar between groups: 31.73 ± 3.78 years in the non-conization group and 31.72 ± 3.88 years in the conization group (P = 0.531). In both groups, the most common age range was 30–34 years. The BMI was slightly lower in the conization group (21.81 ± 3.33) than in the non-conization group (21.99 ± 3.49; P < 0.001). A greater proportion of women in the conization group had a BMI < 18.5 (11.27% vs. 11.00%), whereas a lower proportion had a BMI ≥ 30 (2.73% vs. 3.34%). Comorbidities, including hypertension (3.14% vs. 2.80%, P = 0.007), DM (7.77% vs. 6.36%, P < 0.001), hyperthyroidism (7.46% vs. 6.54%, P < 0.001), and hypothyroidism (11.83% vs. 10.05%, P < 0.001), were significantly more common in the conization group. Similarly, liver disease (32.19% vs. 26.42%), heart disease (27.03% vs. 23.89%), and dyslipidemia (20.60% vs. 15.46%) were more frequently reported in women who underwent conization (all P < 0.001).

**Table 1:** Baseline characteristics of the study population according to conization status.

Characteristic	Non-conization	Conization	P-value
**Age (mean ± SD)**	31.73 ± 3.78	31.72 ± 3.88	0.531
**Younger than 25**	3243 (1.79%)	429 (2.31%)	–
**25–29**	48355 (26.68%)	4993 (26.84%)	–
**30–34**	90002 (49.66%)	9005 (48.41%)	–
**35–39**	33803 (18.65%)	3610 (19.41%)	–
**40 or older**	5821 (3.21%)	565 (3.04%)	–
**BMI (mean ± SD)**	21.99 ± 3.49	21.81 ± 3.33	<0.001
**<18.5**	1927 (11.0%)	2097 (11.27%)	–
**18.5–23**	105274 (58.09%)	11160 (59.99%)	–
**23–25**	25872 (14.28%)	2540 (13.65%)	–
**25–30**	24097 (13.3%)	2297 (12.35%)	–
**≥30**	6054 (3.34%)	508 (2.73%)	–
**Hypertension**	5067 (2.80%)	584 (3.14%)	0.007
**DM**	11521 (6.36%)	1445 (7.77%)	<0.001
**Hyperthyroidism**	11848 (6.54%)	1387 (7.46%)	<0.001
**Hypothyroidism**	18205 (10.05%)	2200 (11.83%)	<0.001
**Liver disease**	47886 (26.42%)	5988 (32.19%)	<0.001
**Heart disease**	43290 (23.89%)	5028 (27.03%)	<0.001
**Dyslipidemia**	28017 (15.46%)	3832 (20.60%)	<0.001
**Autoimmune disease**	2487 (1.37%)	347 (1.87%)	<0.001
**PCOS**	49285 (27.20%)	5987 (32.18%)	<0.001
**Ovarian mass**	11582 (6.39%)	1387 (7.46%)	<0.001
**Myoma**	15149 (8.36%)	1942 (10.44%)	<0.001
**Adenomyosis**	3179 (1.75%)	446 (2.40%)	<0.001
**Missed abortion**	18119 (10.00%)	2004 (10.77%)	<0.001
**Spontaneous abortion**	4515 (2.49%)	517 (2.78%)	0.017

Abbreviations: BMI, body mass index; DM, diabetes mellitus; PCOS, polycystic ovary syndrome; SD, standard deviation.

Gynecological conditions were also more prevalent in the conization group. PCOS (32.18% vs. 27.20%), ovarian masses (7.46% vs. 6.39%), uterine myomas (10.44% vs. 8.36%), and adenomyosis (2.40% vs. 1.75%) were more common (all P < 0.001). Missed abortions (10.77% vs. 10.00%, P < 0.001), spontaneous abortions (2.78% vs. 2.49%, P = 0.017), and autoimmune disease (1.87% vs. 1.37%, P < 0.001) were more prevalent in the conization group.

### Association between cervical conization and adverse pregnancy outcomes

Cervical conization was significantly associated with higher odds of adverse pregnancy outcomes (Table 2).

**Table 2:** Adjusted association between conization and pregnancy outcomes.

Outcome	aOR (95% CI)	P-value
**Gestational hypertension**	1.09 (0.97 − 1.22)	0.165
**Preeclampsia, mild**	0.93 (0.81 − 1.06)	0.276
**Preeclampsia, severe**	0.88 (0.68 − 1.14)	0.342
**Eclampsia**	0.93 (0.57 − 1.51)	0.779
**GDM**	1.10 (1.05 − 1.15)	<0.001
**Cervical incompetence**	3.15 (3.01 − 3.30)	<0.001
**Placenta previa**	1.42 (1.25 − 1.62)	<0.001
**Placenta accreta**	1.41 (1.05 − 1.89)	0.021
**Placenta abruption**	1.11 (0.91 − 1.35)	0.300
**Preterm labor**	1.44 (1.38 − 1.51)	<0.001
**Threatened preterm labor**	1.42 (1.35 − 1.48)	<0.001
**IUGR**	1.15 (1.03 − 1.27)	0.011
**Threatened abortion**	1.18 (1.13 − 1.22)	<0.001
**PPROM**	1.67 (1.52 − 1.83)	<0.001
**Retained placenta**	0.91 (0.83 − 1.00)	0.050
**Polyhydramnios**	0.97 (0.74 − 1.25)	0.800
**Oligohydramnios**	0.99 (0.89 − 1.10)	0.896

Abbreviations: CI, confidence interval; GDM, gestational diabetes mellitus; IUGR, intrauterine growth restriction; aOR, adjusted odds ratio; PPROM, preterm premature rupture of membranes. Values denote adjusted odds ratios (aORs) with 95% CI based on multivariate logistic regression.

**Fig 1:**
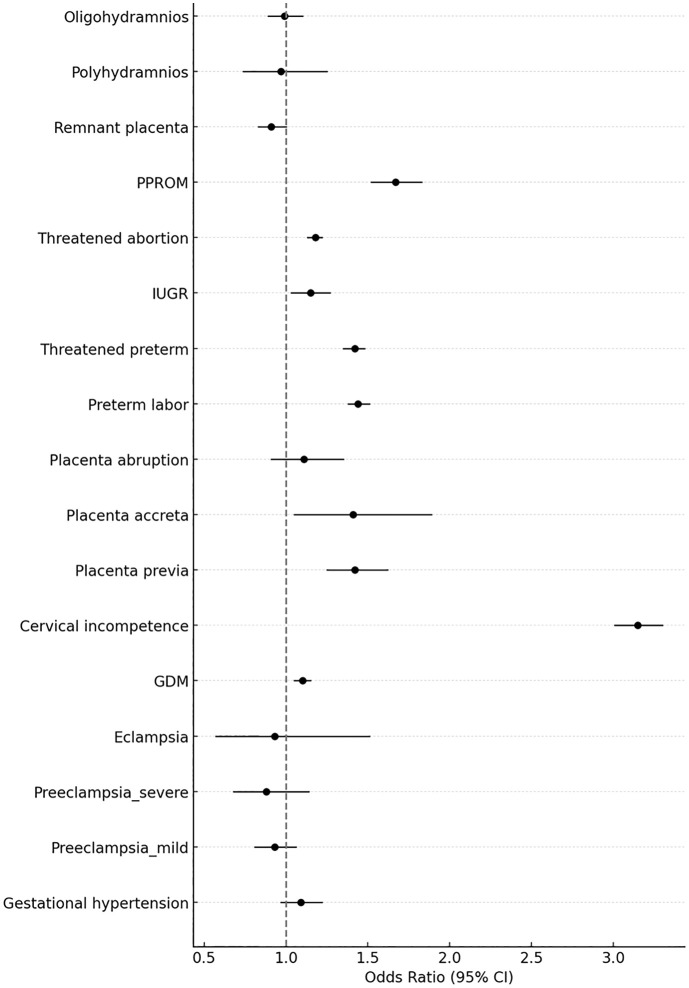
Forest plot of adjusted odds ratios for obstetric complications associated with cervical conization.

Multivariate regression models were adjusted for maternal age, BMI, hypertension, DM, hyperthyroidism, hypothyroidism, PCOS, missed abortions, and spontaneous abortions to account for baseline differences. The strongest association was observed for cervical incompetence (aOR 3.15; 95% CI 3.01–3.30; P < 0.001). Other significantly increased risks included preterm labor (aOR 1.44; 95% CI 1.38–1.51), threatened preterm labor (aOR 1.42; 95% CI 1.35–1.48), and PPROM (aOR 1.67; 95% CI 1.52–1.83), all with P < 0.001. Placental complications were more prevalent in the conization group, including placenta previa (aOR 1.42; 95% CI 1.25–1.62; P < 0.001) and placenta accreta (aOR 1.41; 95% CI 1.05–1.89; P = 0.021). Other statistically significant outcomes included GDM (aOR 1.10; 95% CI 1.05–1.15; P < 0.001), IUGR (aOR 1.15; 95% CI 1.03–1.27; P = 0.011), and threatened abortion (aOR 1.18; 95% CI 1.13–1.22; P < 0.001). The association with retained placenta was borderline (aOR 0.91; 95% CI 0.83–1.00; P = 0.050). No statistically significant associations were observed between gestational hypertension and mild or severe preeclampsia, eclampsia, placental abruption, polyhydramnios, or oligohydramnios. For these outcomes, the aORs were close to 1.0 and the CIs included null values. The forest plot (Fig 1) displays the aORs and 95% CIs for all outcomes.

Forest plot illustrating the adjusted odds ratios (aOR) and 95% confidence interval (CI) for obstetric outcomes comparing women with and without cervical conization. Filled circles and bold lines indicate statistically significant associations (P < 0.05), while open circles and thin lines represent non-significant associations. The vertical dashed line indicates an aOR of 1.0, representing no difference in risk. Abbreviations: GDM, gestational diabetes mellitus; IUGR, intrauterine growth restriction; PPROM, preterm premature rupture of membranes.

### Subgroup analysis: Conization with and without cerclage

Among women with a history of cervical conization, those who underwent cerclage had significantly higher odds of adverse outcomes than did those who did not. These included preterm labor (aOR 4.82; 95% CI 4.32–5.37), threatened preterm labor (aOR 4.67; 95% CI 4.17–5.21), and PPROM (aOR 5.41; 95% CI 4.55–6.43), all with P < 0.001.

In this subgroup, higher risks were also observed for GDM (aOR 1.31; 95% CI 1.15–1.49; P = 0.014) and gestational hypertension (aOR 1.84; 95% CI 1.41–2.40; P = 0.025). Elevated odds were also noted for threatened abortion (aOR 1.76; 95% CI 1.57–1.98). Given that cerclage is typically performed in women at high baseline risk, these elevated odds ratios likely reflect confounding by indication rather than a direct causal effect of the procedure.

Additional outcomes are presented in the Supporting Information (S1 Table and S1 Fig).

## Discussion

This large population-based study demonstrated that cervical conization is associated with an increased risk of adverse obstetric outcomes. The most pronounced association was observed with cervical incompetence, along with significant increases in the risk of preterm labor, PPROM, placenta previa, GDM, and IUGR. These findings are consistent with those reported in previous studies, which collectively suggest that conization may compromise cervical integrity and thereby increase the susceptibility to preterm birth and placental complications [4,6,8,15].

The subgroup analysis further revealed that women who underwent both conization and cerclage exhibited substantially higher risks of preterm labor, threatened preterm labor, and PPROM than did those who underwent conization alone. While this may imply a potential additive effect, it more plausibly reflects the presence of underlying cervical insufficiency or a history of adverse obstetric events, both of which are established indications for cerclage, as noted in the current clinical guidelines [3]. Prior studies have reported elevated risks of adverse pregnancy outcomes after cervical excisional procedures, particularly preterm birth [1,15,16]. Because cerclage is typically offered to women with cervical shortening or a history of spontaneous preterm birth or second-trimester loss [3,6], women receiving post-conization cerclage in observational datasets likely represent a selected high-risk subgroup rather than a group in whom risk is caused by cerclage itself. Therefore, these findings should be interpreted with caution and not be used to directly evaluate the efficacy or harm caused by cerclage. Accordingly, these subgroup estimates are presented as markers of underlying risk selection rather than as causal effects of cerclage. No causal inferences regarding cerclage efficacy or harm can be drawn from our data.

The association between conization and the retained placenta was borderline and should be interpreted with caution. Several outcomes, including polyhydramnios and oligohydramnios, were not statistically significant and are noted in the results for completeness. Although not conclusive, these findings may warrant further evaluation in larger or more detailed datasets.

Pathophysiologically, cervical conization may shorten cervical length, alter the collagen matrix, and compromise the structural and immunological integrity of the cervix. These changes can facilitate premature cervical remodeling and increase vulnerability to ascending infections [15,16]. These effects are supported by studies suggesting that excision procedures may result in cervical stromal damage, inflammatory responses, and potential alterations in the cervical microenvironment [12].

The modest association observed for GDM should be interpreted cautiously given the absence of a clear biologic pathway and the potential for residual confounding in claims-based analyses.

Our findings are consistent with previous systematic reviews and large observational studies reporting an increased risk of preterm birth following cervical excisional procedures [1,16]. Prior literature also suggests that risk may increase with greater excision depth or a larger proportion of cervix removed [1,13]. Because the NHIS claims data do not include excision depth or volume, we could not evaluate whether risk varies by the extent of excision in this cohort. Additionally, this study was strengthened by the inclusion of a large cohort, the use of data reflecting the national population, and the application of standardized diagnostic and procedural codes within a universal health coverage system. The 17-year observation period allowed for robust statistical inference, and the inclusion of a subgroup analysis added granularity to the risk stratification.

However, this study has several limitations. The NHIS database does not include key clinical details such as cervical length, histopathology, indication, excision depth, cone volume, or operative technique at a level that allows reliable stratification by procedure type. These constraints may obscure associations between the extent of excision and risk and dilute differences between techniques. Outcomes were defined from diagnosis and procedure codes, and week-specific gestational age is incompletely captured. As a result, preterm birth could not be reliably evaluated as a separate outcome, and severity-based grading of preterm birth and time-dependent modeling were therefore not feasible. Primary obstetric outcomes strongly predict risk in subsequent pregnancies. Because many details of prior pregnancies are only partially observable in claims data, we prespecified restriction to singleton primiparas to minimize bias from unmeasured obstetric history. Some potential confounders, including antenatal genital tract infections and uterine anomalies, were not included because of limited coding validity, so residual confounding is possible. Not all baseline characteristics in Table 1 were included as covariates to avoid overadjustment and collinearity in a claim setting. Despite these steps, unmeasured confounding cannot be excluded. These structural features of the data source should be considered when interpreting the magnitude and specificity of associations.

These findings underscore the need for careful prenatal surveillance in women with prior cervical procedures and highlight the importance of individualized risk assessment. Future research should aim to further clarify the temporal and mechanistic pathways linking conization to adverse obstetric outcomes and identify women most likely to benefit from enhanced monitoring approaches.

In summary, cervical conization is associated with an increased risk of adverse obstetric outcomes, particularly in women who undergo cervical cerclage. These results should be interpreted in light of selection factors for cerclage and limitations of claims-based outcome definitions and support the need for personalized obstetric care. Prospective studies with detailed clinical and temporal data are warranted to validate and expand these associations.

## Supporting information

S1 Fig Forest plot of adjusted odds ratios for pregnancy complications: Conization only and conization with cerclage. Black circles indicate adjusted odds ratios (aORs) with 95% confidence intervals (CIs) for women who underwent conization alone. Gray triangles represent women who underwent both conization and cervical cerclage. The vertical dashed line at aOR = 1.0 indicates no significant difference in risk. Abbreviations: Gest_htn, gestational hypertension; IUGR, intrauterine growth restriction; PPROM, preterm premature rupture of membranes.
(TIF)

S1 Table Adjusted odds ratios for pregnancy complications in women with conization, with and without cerclage. Comparison of adjusted odds ratios (aORs) and 95% confidence intervals (CI) between women who underwent conization alone and those who underwent both conization and cerclage. Abbreviations: GDM, gestational diabetes mellitus; IUGR, intrauterine growth restriction; PPROM, preterm premature rupture of membranes.
(DOCX)
