# Supplementary figures and images for: Association between cervical conization and pregnancy outcomes: A nationwide population-based cohort study
Source: PLoS One. 2026 Feb 17;21(2):e0341660. doi: 10.1371/journal.pone.0341660 (PMC12912565; doi:10.1371/journal.pone.0341660)

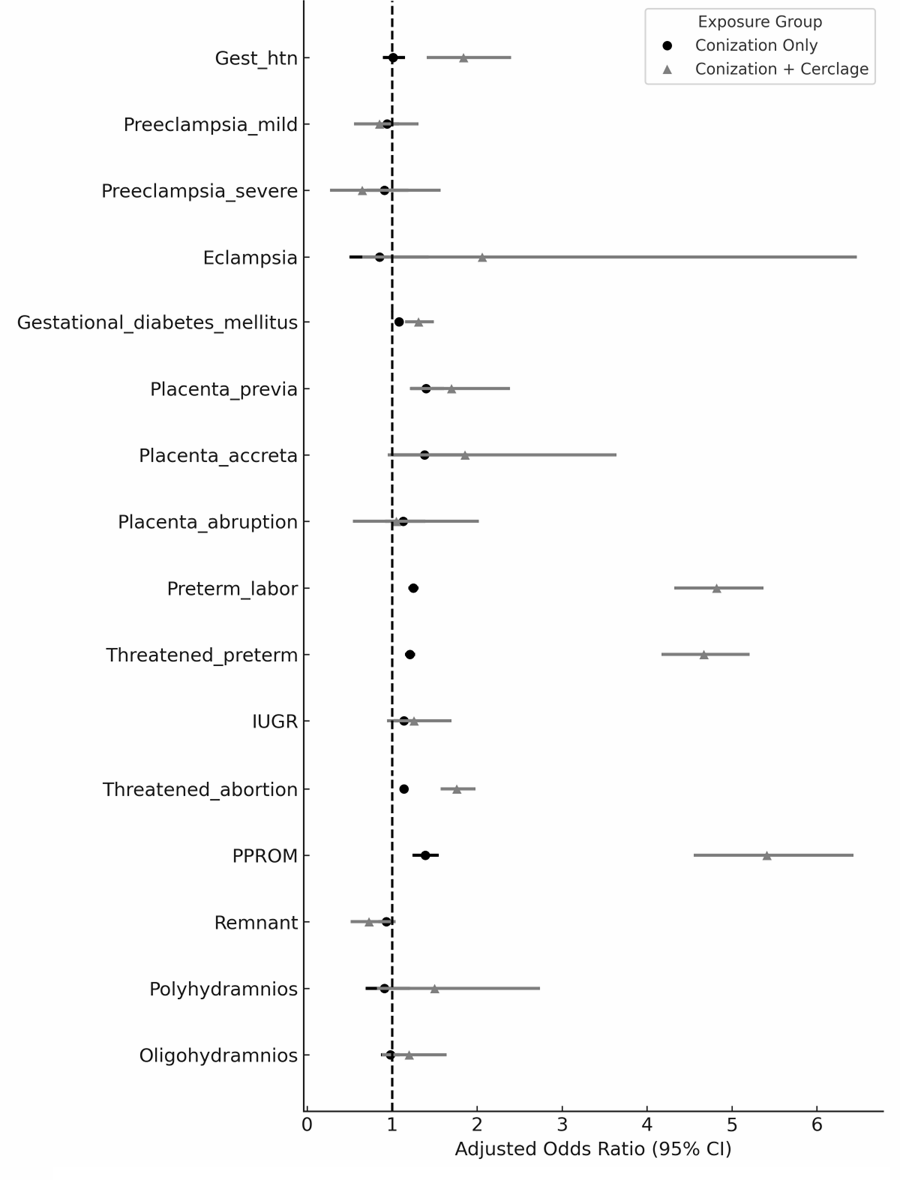

Supplement: S1 Fig — Black circles indicate adjusted odds ratios (aORs) with 95% confidence intervals (CIs) for women who underwent conization alone. Gray triangles represent women who underwent both conization and cervical cerclage. The vertical dashed line at aOR = 1.0 indicates no significant difference in risk. Abbreviations: Gest_htn, gestational hypertension; IUGR, intrauterine growth restriction; PPROM, preterm premature rupture of membranes. (TIF) [file pone.0341660.s001.tif]
