# Supplementary material for: Association between cervical conization and pregnancy outcomes: A nationwide population-based cohort study
Source: PLoS One. 2026 Feb 17;21(2):e0341660. doi: 10.1371/journal.pone.0341660 (PMC12912565; doi:10.1371/journal.pone.0341660)
Supplement: S1 Table — Comparison of adjusted odds ratios (aORs) and 95% confidence intervals (CI) between women who underwent conization alone and those who underwent both conization and cerclage. Abbreviations: GDM, gestational diabetes mellitus; IUGR, intrauterine growth restriction; PPROM, preterm premature rupture of membranes. (DOCX) [file pone.0341660.s002.docx]

**Table S1. Adjusted odds ratios for pregnancy complications in women with conization, with and without cerclage**


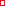


| **Outcome** | **Conization_only aOR (95% CI)** | ***P*_value** | **Conization + Cerclage  aOR (95% CI)** | ***P*_value** |
| --- | --- | --- | --- | --- |
| Gestational hypertension | 1.01 (0.89−1.15) | <0.0001 | 1.84 (1.41−2.40) | 0.025 |
| Preeclampsia, mild | 0.94 (0.81−1.08) | 0.802 | 0.85 (0.55−1.31) | 0.494 |
| Preeclampsia, severe | 0.91 (0.70−1.19) | 0.694 | 0.65 (0.27−1.57) | 0.448 |
| Eclampsia | 0.85 (0.50−1.43) | 0.135 | 2.06 (0.65−6.47) | 0.357 |
| GDM | 1.08 (1.03−1.13) | 0.066 | 1.31 (1.15−1.49) | 0.014 |
| Placenta previa | 1.40 (1.22−1.61) | 0.819 | 1.70 (1.21−2.39) | 0.208 |
| Placenta accreta | 1.38 (1.00−1.89) | 0.325 | 1.86 (0.95−3.64) | 0.595 |
| Placenta abruption | 1.13 (0.92−1.39) | 0.422 | 1.05 (0.54−2.02) | 0.49 |
| Preterm labor | 1.25 (1.19−1.31) | <0.0001 | 4.82 (4.32−5.37) | <0.0001 |
| Threatened preterm labor | 1.21 (1.15−1.27) | <0.0001 | 4.67 (4.17−5.21) | <0.0001 |
| IUGR | 1.14 (1.02−1.28) | 0.099 | 1.26 (0.94−1.70) | 0.93 |
| Threatened abortion | 1.14 (1.09−1.19) | <0.0001 | 1.76 (1.57−1.98) | 0.095 |
| PPROM | 1.39 (1.24−1.55) | <0.0001 | 5.41 (4.55−6.43) | <0.0001 |
| Retained placenta | 0.93 (0.84−1.02) | 0.57 | 0.73 (0.51−1.04) | 0.177 |
| Polyhydramnios | 0.91 (0.69−1.21) | 0.012 | 1.50 (0.82−2.74) | 0.597 |
| Oligohydramnios | 0.98 (0.87−1.09) | 0.018 | 1.20 (0.88−1.64) | 0.746 |

Comparison of adjusted odds ratios (aORs) and 95% confidence interval (CI) between women who underwent conization only and those who underwent both conization and cerclage.

GDM, gestational diabetes mellitus; IUGR, intrauterine growth restriction; PPROM, preterm premature rupture of membranes.
